# The Tetrel Bonds of Hypervalent Halogen Compounds

**DOI:** 10.3390/molecules28207087

**Published:** 2023-10-14

**Authors:** Zhihao Niu, Sean A. C. McDowell, Qingzhong Li

**Affiliations:** 1 The Laboratory of Theoretical and Computational Chemistry, School of Chemistry and Chemical Engineering, Yantai University, Yantai 264005, China; nzh19981019@s.ytu.edu.cn; 2 Department of Biological and Chemical Sciences, The University of the West Indies, Cave Hill Campus, Bridgetown BB11000, Barbados

**Keywords:** tetrel bond, σ-hole, group transfer, hypervalent halogen

## Abstract

The tetrel bond between PhXF_2_Y(TF_3_) (T = C and Si; X = Cl, Br, and I; Y = F and Cl) and the electron donor MCN (M = Li and Na) was investigated at the M06-2X/aug-cc-pVDZ level of theory. As the electronegativity of the halogen atom X increases, the strength of the tetrel bond also increases, but as the electronegativity of the halogen atom Y increases, the strength of the tetrel bond decreases. The magnitude of the interaction energy in most –CF_3_ complexes was found to be less than 10 kcal/mol, but to exceed 11 kcal/mol for PhClF_2_Cl(CF_3_)⋯NCNa. The tetrel bond is greatly enhanced when the –SiF_3_ group interacts with LiCN or NaCN, with the largest interaction energy approaching 100 kcal/mol and displaying a covalent Si⋯N interaction. Along with this enhancement, the Si⋯N distance was found to be less than the X–Si bond length, the –SiF_3_ group to be closer to the N atom, and in most –SiF_3_ systems, the X–Si–F angle to be less than 90°; the –SiF_3_ group therefore undergoes inversion and complete transfer in some systems.

## 1. Introduction

A tetrel bond (TB) is defined as an interaction between a Group 14 element acting as a Lewis acid center and a Lewis base [1]. In recent years, tetrel bonding has become a hot research topic in the fields of materials science, chemistry, and biology [2,3]. Specifically, it has important applications in molecular assembly [4], organic catalysis [5], biological molecular function regulation [6], and so on. When an electron-withdrawing atom or group is covalently-bonded to a Group 14 atom, a positive electrostatic potential region develops on the surface of the tetrel (T) atom. This positive region is dependent on the structure of the Lewis acid itself. When the T atom is sp^3^ and sp^2^ hybridized, such regions are called σ-holes and π-holes, respectively [7,8]. All such regions attract electrons.

The earliest TBs were reported for research on the interactions between methane (with electron-withdrawing substituents) and various nucleophilic reagents. After the H atom on methane is replaced by an F atom, the positive sites generated on the extension of the F-C bond and opposite to the F atom can attract Lewis bases, such as H_2_O [9]. Subsequently, a large amount of crystal data indicates a similar interaction between other Group 14 atoms and Lewis bases [1].

Compared with other congeners, it is generally difficult for C atoms to form TBs, because of their positivity and the low polarizability of their electron clouds [7]. When C atoms participate in TB formation, this interaction is sometimes called a carbon bond [9]. Although the carbon bond is weak, there is still much evidence for its existence [9,10]. For example, when C is adjacent to four strong electron-withdrawing groups (e.g., the –CN group), 1,1,2,2-tetracyanocyclopropane can form a strong carbon bond with tetrahydrofuran, and the interaction energy is 11 kcal/mol [11]. In another study, when the Lewis acid was a cation, the carbon bond was found to be strengthened, resulting in a binding energy of 14–17 kcal/mol [12].

Starting from Si, the strength of the TB increases in the order Si < Ge < Sn. The TB strengths due to Pb and Sn are not significantly different from each other, though their relative stabilities change with the type of base [13]. There is some controversy about their relative magnitudes [14,15], but nevertheless, compared to C, other T atoms are likely to form much stronger TBs. In research by Grabowski on GeF_4_, it was found that when an F atom is replaced by H, its TB strength decreases because the depth of the σ-hole on Ge is reduced [14]. The commonalities between the TB and other weak interactions can also be revealed by charge transfer and AIM methodologies [16]. Later in-depth investigations further extended the findings on TBs. For example, a more recent study by Scheiner on TB strength variation due to systematic replacement of the H atoms of TH_4_ by F atoms found that with the addition of the first three F atoms, the binding energy corresponding to a T⋯N interaction kept rising, but a levelling-off was noticed on addition of the fourth F atom, or even a slight weakening of the binding energy [17]. The molecular electrostatic potential (MEP) maximum on Ge was also determined, from which it was concluded that the sequential addition of F atoms systematically deepened the σ-hole [17].

As noted for the triel bond, which was discovered later [18], the deformation of the monomer also plays an important role in the formation of the TB [19,20,21] and is sometimes so great that the deformation energy causes the computed binding energy to have a positive value [22]. Significant deformation of monomers is often accompanied by charge transfer between molecules [23,24]. The formation of a strong TB is usually accompanied by charge transfer from the lone pair on the Lewis base to a nearby orbital on the Lewis acid [16]. Taking FH_3_Ge as an example, when Ge is replaced by C, the σ*_T-F_ orbital energy increases from 83.46 to 186.37 kcal/mol, thereby reducing the charge-transfer energy and, consequently, the TB strength from 12.7 to 2.16 kcal/mol [25]

TB strength is one of the factors affecting the transfer of –TX_3_ groups. The transfer of TX_3_ groups can be important in some chemical reactions, so some studies have focused on the role of TBs in important chemical processes, especially in S_N_2 reactions. The rotation mechanism of –CH_3_ in the S_N_2 reaction Cl^–^ + CH_3_I was analyzed in detail by molecular beam imaging and chemical kinetics calculations [26]. In this process, a structure similar to a TB complex was found for both reactants and products [26]. Grabowski believed the initial stage of an S_N_2 reaction to be related to TB formation [16]. The latter stage of the reaction N_3_^−^ + CH_3_Br → CH_3_N_3_ also involves the TB [27]. The three-center four-electron structure of carbon ([N–C–N]^+^) is formed by the double-tooth electron donor-capturing carbene ions via a TB, and its structure was found to be similar to the transition state geometry of an S_N_2 reaction [28].

The idea behind the present study of –TX_3_ group transfer was suggested by proton transfer, which is due to hydrogen bonding [29]. Therefore, we speculate that TBs may also cause –TX_3_ transfer. However, it is more difficult to transfer the –TX_3_ group via a TB, as the molecular weight of the –TX_3_ group is large compared to the mass of a proton. The transfer of the –TX_3_ group has, however, been achieved through synergistic effects. For example, the addition of BeCl_2_ molecules to a binary complex via beryllium bonds can enhance the interaction energy; half-transfer of the –GeF_3_ group has been observed [30]. The interaction energy of a TB formed by PhTH_3_ (T = Si, Ge) and a nitrogen heterocyclic carbene (NHC) was found to be less than 4 kcal/mol [31]. When the benzene ring of PhTH_3_ participates in a cation-π interaction with Be^2+^, the interaction energy sharply increases to 100 kcal/mol and complete transfer occurs [31].

Hypervalent halogens have strong oxidizing properties and novel structures compared to monovalent halides, making them important species in organic synthesis [32]. Akiba summarized the general situation regarding the structures and bonding of hypervalent halogens [33]. Hypervalent halogens have also been extensively studied in theoretical research [34,35].

**Scheme 1:**
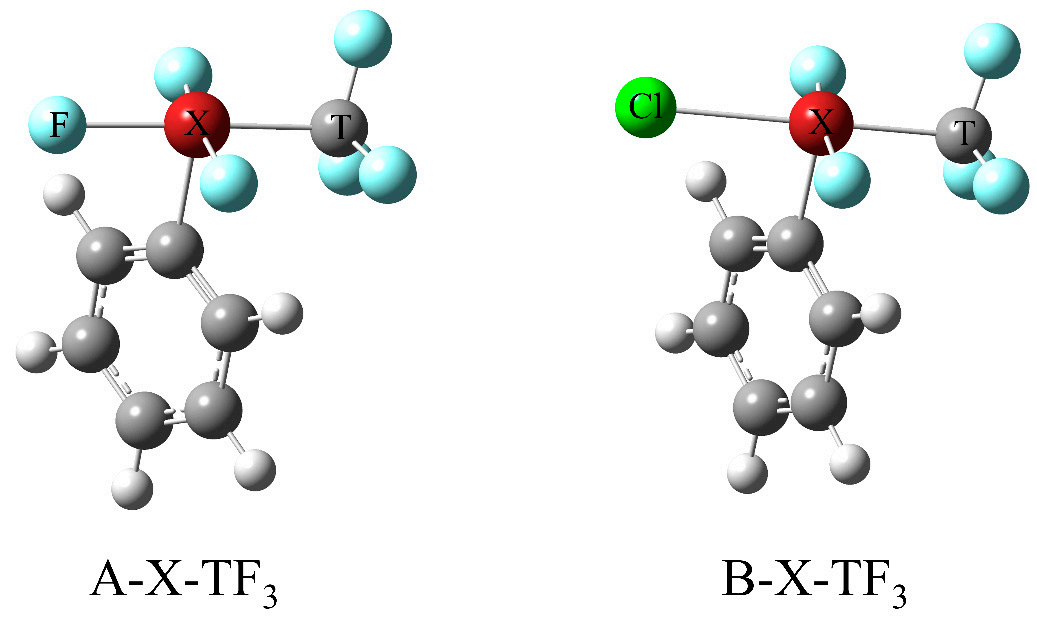
The scheme of A-X-TF_3_ and B-X-TF_3_ (X = F, Cl, Br, I, T = C, Si).

The complete transfer of a –TX_3_ group has been achieved previously through a synergistic effect, and this present work focuses on achieving the complete transfer of the –TX_3_ group without using a third molecule; i.e., without the synergistic effect. We selected the hypervalent halogen compound PhXF_2_Y(TF_3_) as the TB σ-hole donor, where X is a hypervalent halogen atom connected to five atoms or groups; X can be either Cl, Br or I, Ph is a benzene ring, Y is a monovalent halogen atom, and the corresponding molecules are represented as A–XTF_3_ and B–XTF_3_, with A and B representing two situations where Y is either F or Cl, respectively (Scheme 1). MCN (M=Li and Na) are the electron donors. In the present work, the structure and properties of the TB formed between PhXF_2_Y(TF_3_) and MCN, as well as the conditions for the transfer of the –TF_3_ group, are explored and discussed.

## 2. Results

### 2.1. Electrostatic Potentials of Monomers

**Figure 1:**
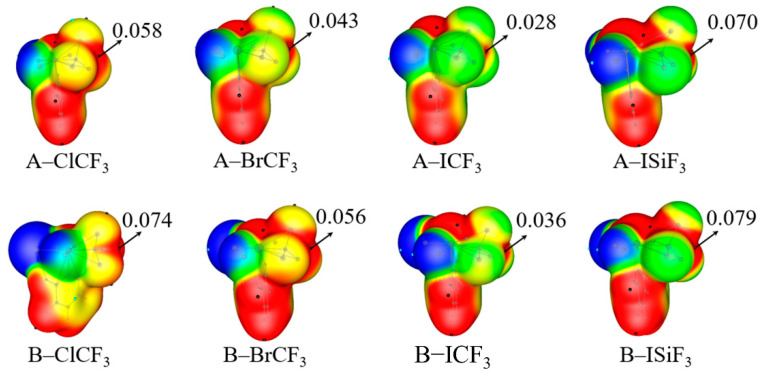
MEP maps of A–XTF_3_ and B–XTF_3_ monomers. Color ranges in a.u are: red, greater than 0.02; yellow, between 0.02 and 0; green, between −0.02 and 0; blue, less than −0.02.

Figure 1 shows the electrostatic potential diagrams for the monomers of the electron acceptors A–XTF_3_ and B–XTF_3_. The red positive electrostatic potential region on the extension line of each molecule’s X–T bond is the σ–hole. The figure also shows the positive electrostatic potential values V_s, max_ on the 0.001 a.u. isosurface. It can be seen from the figure that the V_s, max_ of B–XTF_3_ is greater than that of A–XTF_3_, contrary to the fact that F is more electronegative than Cl (more on this later); however, V_s, max_ decreases in the order X = Cl > Br > I, consistent with the electronegativity of the halogen atom X. The electrostatic potential for the SiF_3_-containing monomer is more than twice the value for its CF_3_-containing analogue.

### 2.2. Geometric Structure and Interaction Energy

**Figure 2:**
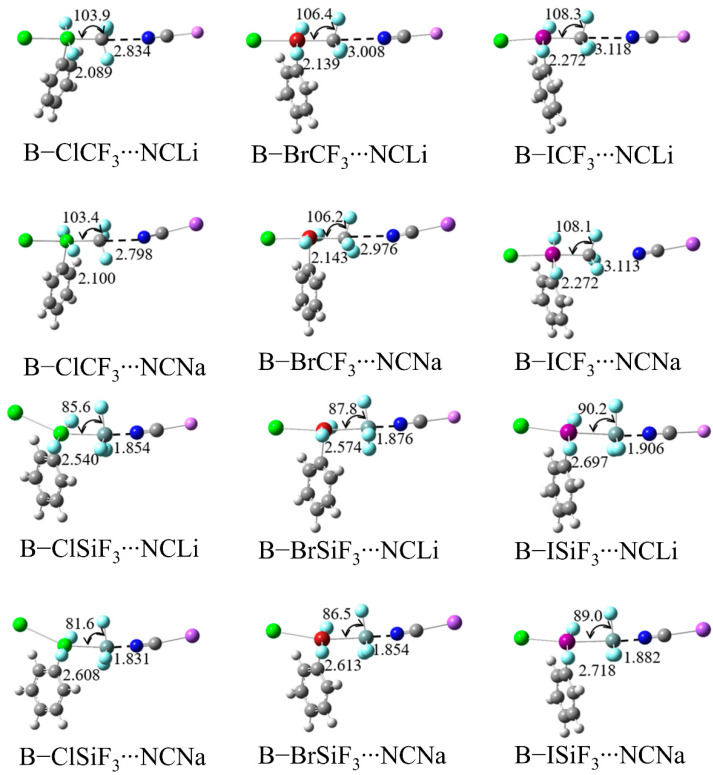
Optimized structures of B–XTF_3_⋯NCM, marked with the mean of the three angles X–T–F (α, deg), T⋯N distance (R_1_, Å) and X–T bond length (R_2_, Å).

Figure 2 shows the structural diagrams of the TB complexes formed by B–XTF_3_ and the electron donor MCN, while the structural diagrams of the A–XTF_3_ systems are shown in Figure S1. Some important geometric parameters of the complexes are listed in Table 1. Firstly, the Si⋯N binding distance is much shorter than that of the C⋯N distance and is represented by R_1_ in the table. The R_1_ values of the SiF_3_ complexes are shorter than 2.0 Å, while the R_1_ values of the CF_3_ complexes are close to or greater than 3 Å. The X–Si distances in the SiF_3_ complexes are longer than the X–C distances in the CF_3_ complexes, represented by R_2_ in the table. The table also shows the difference between R_1_ and R_2_, as well as the change in the X-T bond length on complexation. For the –CF_3_ systems, R_1_ > R_2_, so their difference is positive, indicating that the C⋯N bond is a traditional noncovalent interaction. For the –SiF_3_ systems, R_1_ < R_2_, therefore, the difference is negative, indicating that Si⋯N has become a covalent interaction. The γ_1_ and γ_2_ listed in the table are, respectively, the ratio of R_1_ and R_2_ to the sum of the Van der Waals radii of the two relevant atoms. For the –CF_3_ system, γ_1_ is approximately 0.7, consistent with a weak C⋯N TB, while γ_2_ is about 0.5, reflecting the covalent properties of the X–C bond. For the –SiF_3_ system, γ_1_ is approximately 0.4, while γ_2_ is about 0.5, indicating that the covalent component of the Si⋯N TB is greater than that of the X–Si bond. This indicates that the –SiF_3_ group is located in the middle of the X and N atoms and is closer to the N atom. This indirectly indicates that –SiF_3_ is completely transferred from the X atom of the electron acceptor to the N atom of the electron donor. The last column of Table 1 shows that α(X–T–F) changes from 106° ~ 109° (for the –CF_3_ systems) to values close to 90^o^ (for the –SiF_3_ systems), with some angles significantly smaller than 90^o^, further emphasizing the difference between the tetrel atoms C and Si. It is evident from Figure 2 that the umbrella-like structure of –CF_3_, which is oriented towards N, is transformed into an umbrella-like structure oriented towards X in the –SiF_3_ systems. It is worth noting that α for B–ClSiF_3_⋯NCNa has a relatively small value of 81.6°, indicating the largest transfer.

**Figure 3:**
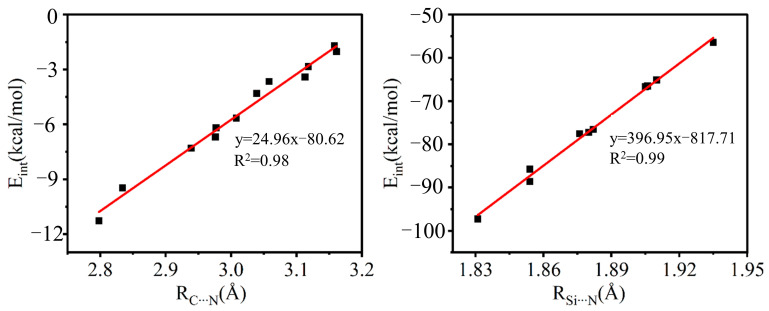
Linear graph of the interaction energy (E_int_) with the T⋯N distance (R_1_) in the complexes of A/B–XCF_3_ (**left**) and A/B–XSiF_3_ (**right**).

The interaction energy (E_int_) of these systems varies widely, from 2 to 97 kcal/mol. E_int_ changes with the electronegativity of the X atom. The larger the electronegativity of X, the larger the value for E_int_; both A–XTF_3_ and B–XTF_3_ show this trend, consistent with the order of the electrostatic potential on the electron acceptor. Similarly, the electron donor NaCN forms a stronger TB than does LiCN, the E_int_ of the –SiF_3_ system is greater than that of the –CF_3_ system, and the TB formed by B–XCF_3_ is stronger than that of A–XCF_3_ (more on this later). The trend for the change in the interaction energy is opposite to the trend for the variation of the T⋯N distance R_1_. Figure 3 intuitively reflects this conclusion. From the graph, we found that E_int_ is correlated with R_1_, with a correlation coefficient as high as 0.98–0.99.

### 2.3. AIM Analysis

**Figure 4:**
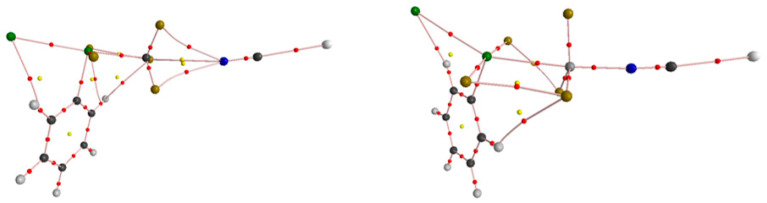
AIM diagrams of B–ClCF_3_⋯NCNa (**left**) and B–ClSiF_3_⋯NCNa (**right**). Bond paths are indicated by broken lines with bond critical points represented by small red dots.

**Figure 5:**
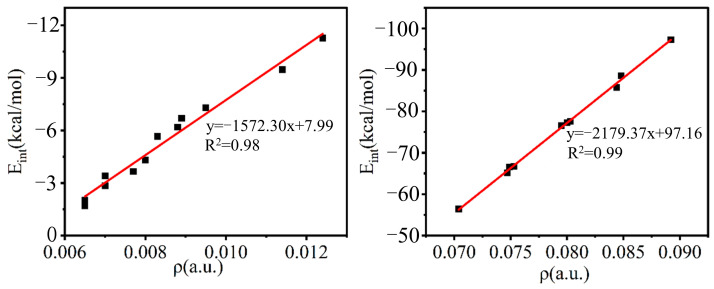
Linear graph of interaction energy (E_int_) and electron density (ρ) at the T⋯N BCP in the complexes of A/B–XCF_3_ (**left**) and A/B–XSiF_3_ (**right**).

Table 2 lists the topological parameters at the bond critical points (BCPs) of T⋯N and X–T, corresponding to the AIM diagrams in Figure 4. These parameters are closely related to the strength of the TB, and Figure 5 shows the relationship between the E_int_ and the electron density ρ at the T⋯N BCP, revealing a strong positive correlation between the parameters; i.e., with an increase in electron density, the interaction energy also increases, with a correlation coefficient of 0.98–0.99. For the CF_3_ complexes, the electron density varies from 0.007 to 0.01 a.u. It can also be seen in Figure 4 that there is no obvious bond path between C and N in C⋯N, but three paths from N to F. For the SiF_3_ complexes, the situation is different. The electron density is one order of magnitude larger than the –CF_3_ value and it can be seen from the figure that there is an obvious bond path between Si and N in the Si⋯N interaction. In addition to the numerical changes, we also noticed a change in sign. When the C atom is replaced by the Si atom, the energy density H at the T⋯N BCP also changes from positive to negative, indicating a change in the type of T⋯N bond from noncovalent interactions in the –CF_3_ systems to partially covalent interactions in the –SiF_3_ systems, resulting in an enhanced TB.

The X–C covalent bond has large electron density values (greater than 0.1 a.u.) and a large Laplacian and total energy density. The ρ at the X–C BCP is approximately 0.1 a.u., ∇^2^ρ < 0, H < 0, which indicates that the X–C bond is a typical covalent bond. For the –SiF_3_ system, the electron density at the BCP of the X–Si bond is lower than that of the Si–N bond, indicating that the Si–N bond is more tightly bound than the X–Si bond, which is consistent with the point that the covalent contribution to Si–N is greater than in the X–Si bond, as mentioned earlier. ∇^2^ρ > 0, H < 0 indicates that the Si–N bond is a partially covalent interaction. There are exceptions, as when X = I, the ∇^2^ρ at the BCP of the X–Si bond is also negative, but this is still a typical covalent bond.

**Figure 6:**
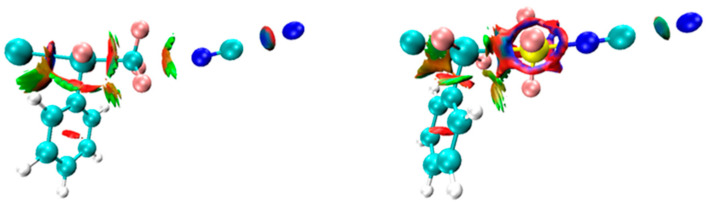
NCI diagram of B–ClCF_3_∙∙∙NCNa (**left**) and B–ClSiF_3_∙∙∙NCNa (**right**). Blue, green, and red areas represent strong attraction, weak attraction, and strong repulsion interactions, respectively.

Figure 6 is the NCI diagram of the B–XTF_3_⋯NCM system, with an orange-green area sandwiched between C and N that represents the medium strength C⋯N tetrel bond. There is a blue region along the Si⋯N bond axis that represents a strong attraction, surrounded by a red region representing repulsion, which indicates overall a strong Si⋯N TB.

### 2.4. NOCV Analysis

Table 3 shows the charge transfer (CT) between the interacting monomers of the complex, as well as the change in the charge on the A–X or B–X molecular subunits. For the –CF_3_ system, the CT value is relatively small, ranging from 0.002 to 0.007 e; This parameter increases significantly in the –SiF_3_ system, with most values now in the vicinity of 0.2 e. For the B–ClSiF_3_⋯NCM system, the charge transfer exceeds one electron, which is consistent with the change in the interaction energy of these systems.

**Figure 7:**
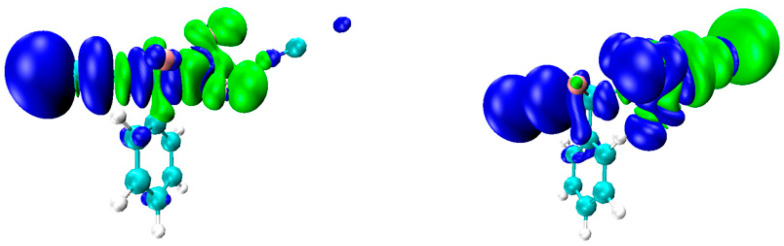
NOCV pair density map of B–ClCF_3_⋯NCNa (**left**) and B–ClSiF_3_⋯NCNa (**right**).

The formation of TBs can also be analyzed using the natural orbital for chemical valence (NOCV) method. The NOCV density contour map is shown in Figure 7, which is composed of blue and green contour maps, where blue represents electrons accepted and green represents electrons donated. The area near the N atom is green, while the area near –TF_3_ is blue, which means that the electron density is transferred from the lone-pair electron orbital of the N atom into the antibonding molecular orbital of X–T. The energy corresponding to this orbital interaction is also given in Table 3.

We found a good linear relationship between the NOCV orbital energy and the interaction energy. Figure S2 shows the linear relationship between these parameters in the –SiF_3_ system, with a correlation coefficient of 0.95. The orbital effect in the –CF_3_ systems is not significant, with the most negative NOCV energy being −0.81 kcal/mol, which is negligible and can therefore be ignored. On the other hand, the orbital effects in the –SiF_3_ systems are significant, with NOCV energies ranging between −54.86 and −82.60 kcal/mol.

**Figure 8:**
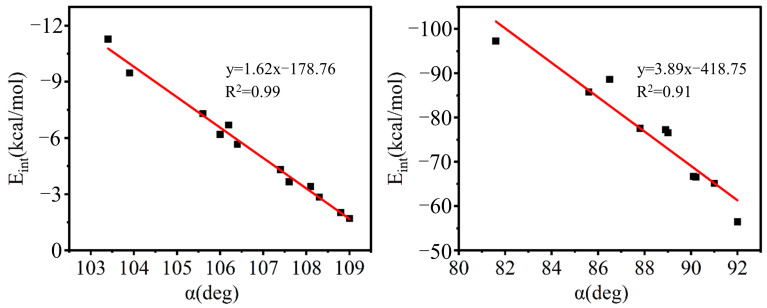
Linear relationship between the interaction energy (E_int_) and the X–T–F angle (α) in the complexes of A/B–XCF_3_ (**left**) and A/B–XSiF_3_ (**right**).

Presumably, the orbital interaction and charge transfer are also facilitated by the previously mentioned change in α(X-T-F) from more to less acute values, consistent with the strong linear correlation between E_int_ and α shown in Figure 8 above. 

### 2.5. Energy Decomposition Analysis

The total interaction energy of the complex is decomposed into electrostatic energy (E^es^), exchange energy (E^ex^), repulsion energy (E^rep^), polarization energy (E^pol^), and dispersion energy (E^disp^) contributions. The relevant data are presented in Table 4. From the table, it can be seen that the electrostatic energy makes the largest contribution to the attractive energies for most systems. For most –CF_3_ systems, in addition to the electrostatic energy, the contributions from the dispersion energy and the exchange energy are also relatively large, while the contribution from the polarization energy is the smallest. For the –SiF_3_ system, these energies increase significantly by several orders of magnitude. In addition to the electrostatic energy, the contribution from the polarization energy is also very important. For example, the polarization energy of the B–ClSiF_3_⋯NCNa system is as high as 108.94 kcal/mol. The most surprising observation is that the NOCV orbital energy of the B–ClSiF_3_⋯NCNa system is also very high, attaining a value of 82.60 kcal/mol. Unlike the –CF_3_ system, the dispersion energy makes the smallest contribution to the interaction energy in the –SiF_3_ systems, though its value is still quite large. The relationship between the total interaction energy and three attractive terms in the –SiF_3_ system (E^es^, E^pol^, and E^disp^) were plotted and the three parameters are linearly related to the total interaction energy, as shown in Figure S3. The dispersion energy, which hardly changes with a change in the total interaction energy, is depicted in the figure by what is essentially a horizontal straight line. The relationship between the electrostatic energy and the total interaction energy is similar to the relationship between the polarization energy and the total interaction energy, as the slopes of their straight lines are similar.

## 3. Computational Methodology

All calculations were performed at the M06-2X/aug-cc-pVDZ [36,37] level of theory using the Gaussian 09 suite of programs [38], because this method can provide a reliable description of noncovalent bonding in molecular systems, including complexes held together by tetrel bonds [39,40]. In order to ensure that all structures are real minima on their respective potential energy surfaces, vibrational frequency calculations were undertaken at the same level, thereby confirming that there were no imaginary frequencies. The interaction energies were calculated using the supermolecule method, and the basis set superposition error (BSSE) correction was performed using the counterpoise method proposed by Boys and Bernardi [41].

To predict the interaction sites and their magnitudes, wave function surface analysis software (WFA-SAS) [42] was used to calculate the molecular electrostatic potential (MEP) of each monomer and binary complex on the 0.001 a.u. isosurface. The electron density, its Laplacian and total energy density at the bond critical point (BCP), were analyzed by using Bader’s atoms in molecules (AIM) theory [43] and the Multiwfn program [44]. Natural bond orbital (NBO) analyses were performed using NBO 5.0 software [45]. Multiwfn [44] and VMD software [46] were used for natural orbitals for chemical valence (NOCV) [47] and noncovalent interaction (NCI) analyses, and for drawing their diagrams. The localized molecular orbital energy decomposition analysis (LMO-EDA) [48] implemented in the GAMESS suite of programs [49] was used to partition the interaction energy into five different energy components: electrostatic energy, exchange energy, repulsion energy, polarization energy, and dispersion energy.

## 4. Discussion

Previous studies found that the TB formed by the –CF_3_ group is weak, and even when the electron donor atom is connected to hydrogen atoms, it is more inclined to form hydrogen bonds. When anions act as electron donors in these types of complexes, their interaction energy rarely exceeds 7 kcal/mol [50]. However, when the –CF_3_ group is connected to a hypervalent halogen group, it not only forms a TB but also gives rise to relatively large interaction energies in many cases, such as in B–ClCF_3_⋯NCNa, where the interaction energy exceeds 11 kcal/mol. Therefore, we have found an effective way in the present work to promote strong TB formation with the –CF_3_ group.

The influence on the TB of the halogen atom X connected to the –TF_3_ group should not be underestimated, since the strength of the TB increases in the order X = I < Br < Cl. The greater the electronegativity of the X atom, the stronger the TB, since the electron-withdrawing ability of X increases accordingly and, consequently, deepens the σ–hole. This effect on the TB is similar to the effect of the halogen atom X in XTF_3_ [51].

However, when the halogen atom Y on the electron acceptor PhXF_2_Y(TF_3_) changes from Cl to F, the depth of the σ–hole decreases and the corresponding TB weakens, which is inconsistent with the relative electronegativities of the Y halogen atoms. This puzzling finding can be rationalized by considering two similar model complexes, denoted for clarity as Y-FClF_2_-(CF_3_)∙∙∙NCLi, where Y=F or Cl, X=Cl, T=C and F replaces the Ph group in the electron acceptor PhXF_2_Y(TF_3_). The optimized geometries for these model complexes are similar to the structures shown in Figure S1 (corresponding to A–ClCF_3_⋯NCLi; Y=F) and Figure 2 (corresponding to B–ClCF_3_⋯NCLi; Y=Cl), except that the phenyl group, perpendicular to the Y-Cl-C axis, has been replaced by an F atom. The complexes, with and without the phenyl group, have similar computed properties, with the Y=Cl systems being more stable than the Y=F systems.

With reference to Scheme 1, Figure 1, Figure 2 and Figure S1, the electron acceptor B–Cl–CF_3_ (Y=Cl) has a larger dipole moment than A–Cl–CF_3_ (Y = F) because the F atom opposite to C, on the extension of the Cl–C axis, gives rise to a larger Cl positive charge in A–Cl–CF_3_ than the positive charge on the corresponding Cl atom in B–Cl–CF_3_ when this F atom is replaced by Cl; the M062x/6-31++G(d,p) NBO charges for Cl are +1.676 (Y=Cl) and +1.428e (Y=F), for the electron acceptors with Ph replaced by F. Since the other F atoms perpendicular to the F−Cl−C or Cl−Cl−C axis were found to have similar negative NBO charges in both molecules (ranging between 0.4 and 0.45*e* in magnitude), the negative charge on the B-ClF_3_ subunit (*q* = −0.136; Y = Cl) is larger than the negative charge on the A-ClF_3_ subunit (*q* = −0.073; Y = F). Hence, the larger charge separation between the negatively-charged B-ClF_3_ subunit and the positively-charged CF_3_ subunit in B–Cl–CF_3_, along with a longer R_2_ (i.e., Cl-C bond length, see Table 1), gives rise to a dipole moment larger than the A–Cl–CF_3_ dipole moment. The larger V_s, max_ values in Figure 1 for the B-XTF_3_ monomers, compared to the A-XTF_3_ monomers, are also consistent with this explanation.

The larger B–Cl–CF_3_ dipole thus yields more strongly bound complexes with NCLi than does A–Cl–CF_3_, with the intermolecular interaction dominated by dipole–dipole electrostatic forces (see Table 4), especially since the intermolecular charge transfer for both complexes is small (CT < 0.007 e, see Table 3).

On complexation, the electric field due to the NCLi dipole shifts charge from the CF_3_ subunit into the Cl–C internuclear region, leading to Cl–C bond extension, a more positive CF_3_ subunit and a more negative A–Cl or B-Cl subunit. This, in turn, leads to an increase in the net dipole moment (relative to the isolated monomers), which stabilizes the interaction with NCLi to a greater extent in B–Cl–CF_3_⋯NCLi than in A–Cl–CF_3_⋯NCLi. Table 1 shows the larger Cl–C bond extension due to complexation (ΔR_2_) for the B-ClCF_3_ complex (relative to the A–ClCF_3_ complex), while Table 3 shows the larger increase in the negative charge on the B–Cl subunit due to complexation (relative to the A-Cl subunit). For T = Si, the significantly larger charge transfer (CT > 0.19 e, Table 3) and polarization (Table 4) in the SiF_3_ complexes augment the electrostatic forces, leading to complexes that are much more strongly bound than their CF_3_ counterparts (see Table 1 and Table 4). 

The impact of orbital interactions on TB strength thus cannot be ignored. According to the NOCV results, the orbital interaction between the molecules is mainly due to the charge transfer from the lone pair electrons on N into the antibonding molecular orbital of T–X. As mentioned above, the orbital interactions in the –CF_3_ systems are not significant, while the –SF_3_ systems involve significant orbital interactions. The polarization energy components obtained from the energy decomposition analysis also indirectly confirm this observation. The difference between orbital interactions in the –SF_3_ and –CF_3_ systems plays a decisive role in the differences between their interaction energies. The EDA analysis is also consistent with this finding. In the –CF_3_ systems, the polarization energy is relatively small and does not exceed −4.69 kcal/mol, which suggests minor orbital interactions. On the contrary, in the –SiF_3_ systems, the polarization energy has grown significantly and is now more comparable in magnitude to even the electrostatic energy.

In this work, a hypervalent halogen group is adjacent to –TF_3_, while in the XTF_3_ molecule, the –TF_3_ group is adjacent to a monovalent halogen atom X. It is therefore of interest to compare the strengths of the TBs formed in these two different situations. The interaction energy of the ClSiF_3_⋯NCLi system is approximately 2 kcal/mol [52] and the interaction energy of the FSiF_3_⋯NCLi system is 8 kcal/mol [16], whereas the interaction energy of the B–ClSiF_3_⋯NCLi system is 85 kcal/mol, which is significantly larger than for the first two systems. The most positive electrostatic potential of the C atom in ClCF_3_ is 0.041 a.u., while the most positive electrostatic potentials of the C atom in A–ClCF_3_ and B–ClCF_3_ are 0.058 a.u. and 0.074 a.u., respectively, which are both larger in magnitude than the former. Therefore, adding a hypervalent halogen functional group to –TF_3_ makes it a better σ–hole donor, and it thus forms a stronger TB.

The electron donor used in this work is MCN, where M is an alkali metal. Alkali metal atoms are prone to losing electrons, so when the H atom in HCN is replaced by an alkali metal, the corresponding molecule becomes a stronger electron donor and therefore forms a stronger TB. Such alkali metal substituents can also make methyl C atoms nucleophilic [53]. If the electron donor has more than one alkali metal substituent, its electron-donating ability increases and the resulting TB is further strengthened. For example, the interaction energy between CH_3_F and C_2_H_2_ is only 1 kcal/mol, but two Li/Na substituents increase this amount to 5–6 kcal/mol [54]. Of course, other metals have similar enhancement effects. For example, the TB formed between HCN and SiF_4_ is weak, with an interaction energy of only 3.45 kcal/mol. However, if HCN is replaced by AgCN, the interaction energy of the TB increases nearly fourfold [55].

In our previous work on tetrel bonds, the same electron donor MCN was used [56], but the strength of the TB formed and the degree of –SiF_3_ group transfer are different from the corresponding results from the present study. The interaction energy of the TA–SiF_3_⋯NCNa (TA = tetrazole) system is 53 kcal/mol. When BH_3_ or BF_3_ is added to different N atoms of TA to form a triel bond, the interaction energy of the tetrel bond in the ternary complex increases to 60–66 kcal/mol. The interaction energy of the A/B–XSiF_3_⋯NCNa system can reach 56–97 kcal/mol in magnitude. Therefore, the TB formed by the –TF_3_ group connected to a hypervalent halogen atom is generally stronger than the TB formed by the –TF_3_ group connected to an N atom. The most positive electrostatic potential of Si in the A/B–XSiF_3_ molecule is greater than 0.07 a.u., while it is 0.11 a.u. in the TA–SiF_3_ molecule. However, the former forms a stronger TB, suggesting that deformation plays an important role in the formation of the former.

The α value in the TA–SiF_3_⋯NCNa system is 93°, dropping to 91° in the ternary complex, and the overall structure is triangular biconical, with two N atoms occupying the top position of the triangular biconical. Thus the –SiF_3_ group achieves a half-transfer from tetrazole to NaCN. In A/B–XSiF_3_⋯NCNa, the range of α is 81–92°. In some systems, the –SiF_3_ group is closer to NaCN, achieving complete transfer of the –SiF_3_ group. By utilizing the synergistic effect of cation-π interactions, this group can also achieve half-transfer from the C atom of the benzene ring to the C atom of a nitrogen heterocyclic carbene [31]. In conventional molecules, the bonding energy of the Si–N bond is similar to that of the Cl–Si bond, but in the hypervalent halide compounds, the bonding energy of the latter is lower than in conventional halide compounds. Therefore, the –SiF_3_ group in A/B–XSiF_3_ molecules is prone to transfer. The bonding energy of the Si–O bond is greater than that of the Si–N bond, which suggests that if the electron donor MCN is replaced by pyridine oxide, the –SiF_3_ group in A/B–XSiF_3_ systems is more likely to undergo transfer, and the extent of transfer likely to be greater. In previous work [57], we achieved half-transfer of SiF_3_ using the substituent effect; in the present study, we have demonstrated the full transfer of SiF_3_ without cooperative or substitution effects.

## 5. Conclusions

The TBs formed between PhXF_2_Y(TF_3_) and MCN were investigated at the M06-2X/aug-cc-pVDZ level of theory. When –TF_3_ is adjacent to a hypervalent halogen atom, the TB formed increases with the electronegativity of the hypervalent halogen atom X and decreases with the electronegativity of the halogen atom Y; both C and Si exhibit this behavior. For the –CF_3_ systems, the interaction energy ranges in magnitude between 1 and 12 kcal/mol, forming medium strength C⋯N TBs. For the –SiF_3_ systems, the interaction energy can become as large as 56–97 kcal/mol in magnitude, forming strong Si⋯N TBs. Electrostatic interactions play a dominant role in the formation of T⋯N TBs. In the –SiF_3_ systems, orbital interactions are more pronounced than in the –CF_3_ systems, and even comparable in magnitude to electrostatic interactions. The orbital interaction between the molecules is mainly due to the charge transfer from the lone-pair electrons on N into the antibonding molecular orbital of T–X. The C⋯N TBs are largely closed-shell interactions, whereas the Si⋯N TBs are characterized by partially covalent interactions. For the –SiF_3_ systems, the Si⋯N interaction distance is shorter than the X–Si bond length, and the –SiF_3_ group is more inclined towards the N atom than the X atom, with α values in most of the –SiF_3_ systems found to be less than 90°, indicating inversion and complete transfer of the –SiF_3_ group.

## Figures and Tables

**Table 1:** Interaction energy (E_int_, kcal/mol), T⋯N distance (R_1_, Å), X–T bond length (R_2_, Å) and its change on complexation (ΔR_2_, Å), and average of the three X–T–F angles (α, deg) in the complexes.

	E_int_	R_1_	R_2_	R_1_–R_2_	ΔR_2_	γ_1_^a^	γ_2_^a^	α
A–ClCF_3_⋯NCLi	−6.19	2.977	2.012	0.965	0.034	0.750	0.490	106.0
A–ClCF_3_⋯NCNa	−7.30	2.939	2.017	0.922	0.039	0.740	0.491	105.6
A–BrCF_3_⋯NCLi	−3.66	3.058	2.097	0.961	0.012	0.770	0.492	107.6
A–BrCF_3_⋯NCNa	−4.31	3.039	2.099	0.940	0.014	0.765	0.493	107.4
A–ICF_3_⋯NCLi	−1.70	3.158	2.253	0.905	0.000	0.795	0.512	109.0
A–ICF_3_⋯NCNa	−2.02	3.161	2.254	0.907	0.001	0.796	0.512	108.8
B–ClCF_3_⋯NCLi	−9.47	2.834	2.089	0.745	0.057	0.714	0.508	103.9
B–ClCF_3_⋯NCNa	−11.27	2.798	2.100	0.698	0.068	0.705	0.511	103.4
B–BrCF_3_⋯NCLi	−5.66	3.008	2.139	0.869	0.02	0.758	0.502	106.4
B–BrCF_3_⋯NCNa	−6.69	2.976	2.143	0.833	0.024	0.750	0.503	106.2
B–ICF_3_⋯NCLi	−2.84	3.118	2.272	0.846	0.004	0.785	0.503	108.3
B–ICF_3_⋯NCNa	−3.41	3.113	2.272	0.841	0.004	0.784	0.516	108.1
A–BrSiF_3_⋯NCLi	−66.69	1.905	2.508	−0.603	--	0.451	0.556	90.1
A–BrSiF_3_⋯NCNa	−77.25	1.880	2.535	−0.655	--	0.445	0.562	88.9
A–ISiF_3_⋯NCLi	−56.43	1.935	2.656	−0.721	0.09	0.459	0.571	92.0
A–ISiF_3_⋯NCNa	−65.13	1.910	2.673	−0.763	0.107	0.453	0.575	91.0
B–ClSiF_3_⋯NCLi	−85.74	1.854	2.540	−0.686	--	0.439	0.583	85.6
B–ClSiF_3_⋯NCNa	−97.27	1.831	2.608	−0.777	--	0.434	0.598	81.6
B–BrSiF_3_⋯NCLi	−77.57	1.876	2.574	−0.698	0.199	0.445	0.571	87.8
B–BrSiF_3_⋯NCNa	−88.60	1.854	2.613	−0.759	0.238	0.439	0.579	86.5
B–ISiF_3_⋯NCLi	−66.53	1.906	2.697	−0.791	0.11	0.452	0.580	90.2
B–ISiF_3_⋯NCNa	−76.56	1.882	2.718	−0.836	0.131	0.446	0.585	89.0

^a:^ γ is the ratio of R_1_ or R_2_ and the sum of the two atoms’ van der Waals radius.

**Table 2:** AIM electron density (ρ), its Laplacian (∇^2^ρ) and total energy density (H) at the T⋯N and X–T BCPs in the binary complexes; all quantities are in a.u.

	T⋯N ^a^			X–T		
	ρ	∇^2^ρ	H	ρ	∇^2^ρ	H
A–ClCF_3_⋯NCLi	0.0088	0.0456	0.0022	0.1393	−0.1276	−0.0735
A–ClCF_3_⋯NCNa	0.0095	0.0491	0.0023	0.1372	−0.1207	−0.0715
A–BrCF_3_⋯NCLi	0.0077	0.0409	0.0021	0.1387	−0.1689	−0.0784
A–BrCF_3_⋯NCNa	0.0080	0.0420	0.0021	0.1381	−0.1673	−0.0777
A–ICF_3_⋯NCLi	0.0065	0.0347	0.0018	0.1171	−0.0795	−0.0614
A–ICF_3_⋯NCNa	0.0065	0.0344	0.0018	0.1172	−0.0818	−0.0615
B–ClCF_3_⋯NCLi	0.0114	0.0575	0.0025	0.1142	−0.0480	−0.0509
B–ClCF_3_⋯NCNa	0.0124	0.0608	0.0024	0.1108	−0.0388	−0.0481
B–BrCF_3_⋯NCLi	0.0083	0.0437	0.0022	0.1252	−0.1196	−0.0641
B–BrCF_3_⋯NCNa	0.0089	0.0464	0.0022	0.1241	−0.1163	−0.0629
B–ICF_3_⋯NCLi	0.0070	0.0372	0.0019	0.1132	−0.0801	−0.0568
B–ICF_3_⋯NCNa	0.0070	0.0381	0.0021	0.1134	−0.0823	−0.0570
A–BrSiF_3_⋯NCLi	0.0753	0.3637	−0.0148	0.0589	0.0156	−0.0300
A–BrSiF_3_⋯NCNa	0.0800	0.4014	−0.0156	0.0554	0.0124	−0.0276
A–ISiF_3_⋯NCLi	0.0704	0.3240	−0.0141	0.0664	−0.0435	−0.0384
A–ISiF_3_⋯NCNa	0.0747	0.3576	−0.0147	0.0638	−0.0404	−0.0364
B–ClSiF_3_⋯NCLi	0.0844	0.4452	−0.0156	0.0384	0.0280	−0.0145
B–ClSiF_3_⋯NCNa	0.0892	0.4855	−0.0165	0.0333	0.0270	−0.0110
B–BrSiF_3_⋯NCLi	0.0803	0.4080	−0.0151	0.0498	0.0085	−0.0237
B–BrSiF_3_⋯NCNa	0.0848	0.4444	−0.0160	0.0458	0.0066	−0.0208
B–ISiF_3_⋯NCLi	0.0749	0.3636	−0.0144	0.0598	−0.0391	−0.0330
B–ISiF_3_⋯NCNa	0.0795	0.3995	−0.0151	0.0568	−0.0355	−0.0305

^a:^ The topological parameter of the BCP between CF3 complex molecules is the sum of three F⋯T.

**Table 3:** Charge transfer between A/B–XTF_3_ and MCN (CT, e), NOCV orbital energies (E, kcal/mol) and the change in the charge on the A–X or B–X molecular subunit (Δq, e).

	CT	E	Δq
A–ClCF_3_⋯NCLi	0.0049	−0.54	−0.1121
A–ClCF_3_⋯NCNa	0.0049	−0.65	−0.1289
A–BrCF_3_⋯NCLi	0.0064	−0.44	−0.0844
A–BrCF_3_⋯NCNa	0.0065	−0.52	−0.0950
A–ICF_3_⋯NCLi	0.0069	−0.39	−0.0656
A–ICF_3_⋯NCNa	0.0069	−0.46	−0.0743
B–ClCF_3_⋯NCLi	0.0033	−0.67	−0.1630
B–ClCF_3_⋯NCNa	0.0023	−0.81	−0.1890
B–BrCF_3_⋯NCLi	0.0058	−0.55	−0.1093
B–BrCF_3_⋯NCNa	0.0058	−0.67	−0.1242
B–ICF_3_⋯NCLi	0.0067	−0.46	−0.0819
B–ICF_3_⋯NCNa	0.0068	−0.55	−0.0919
A–BrSiF_3_⋯NCLi	0.2038	−59.35	--
A–BrSiF_3_⋯NCNa	0.2167	−64.21	--
A–ISiF_3_⋯NCLi	0.1890	−54.86	−0.3051
A–ISiF_3_⋯NCNa	0.2017	−59.38	−0.3316
B–ClSiF_3_⋯NCLi	0.2268	−73.27	--
B–ClSiF_3_⋯NCNa	0.2379	−82.60	--
B–BrSiF_3_⋯NCLi	0.2167	−65.42	−0.3282
B–BrSiF_3_⋯NCNa	0.2287	−71.74	−0.3612
B–ISiF_3_⋯NCLi	0.2026	−59.63	−0.3553
B–ISiF_3_⋯NCNa	0.2148	−64.52	−0.3882

**Table 4:** Electrostatic (E^es^), exchange (E^ex^), repulsion (E^rep^), polarization (E^pol^), and dispersion energies (E^disp^), as well as the total interaction energy (E_total_), for the complexes; all energies in kcal/mol.

	E^es^	E^ex^	E^rep^	E^pol^	E^disp^	E_total_
A–ClCF_3_⋯NCLi	−8.36	−4.17	13.73	−2.03	−5.34	−6.17
A–ClCF_3_⋯NCNa	−9.82	−4.90	15.69	−2.44	−5.77	−7.23
A–BrCF_3_⋯NCLi	−5.50	−3.62	12.06	−1.77	−4.90	−3.73
A–BrCF_3_⋯NCNa	−6.25	−4.03	13.11	−2.07	−5.12	−4.35
A–ICF_3_⋯NCLi	−2.93	−7.04	11.85	−0.93	−2.66	−1.71
A–ICF_3_⋯NCNa	−3.15	−2.89	10.17	−1.78	−4.38	−2.03
B–ClCF_3_⋯NCLi	−12.72	−12.45	22.10	−4.69	−1.56	−9.33
B–ClCF_3_⋯NCNa	−14.88	−6.66	20.85	−3.62	−6.72	−11.03
B–BrCF_3_⋯NCLi	−7.51	−3.93	13.00	−2.18	−5.03	−5.65
B–BrCF_3_⋯NCNa	−8.74	−4.54	14.63	−2.59	−5.37	−6.62
B–ICF_3_⋯NCLi	−4.18	−2.99	10.61	−1.78	−4.50	−2.85
B–ICF_3_⋯NCNa	−4.67	−3.19	11.10	−2.07	−4.59	−3.42
A–BrSiF_3_⋯NCLi	−94.92	−56.18	171.44	−70.13	−20.35	−70.14
A–BrSiF_3_⋯NCNa	−104.78	−59.61	181.87	−77.47	−20.76	−80.75
A–ISiF_3_⋯NCLi	−87.57	−55.70	169.53	−61.97	−20.73	−56.44
A–ISiF_3_⋯NCNa	−96.58	−59.36	180.35	−68.34	−21.20	−65.13
B–ClSiF_3_⋯NCLi	−116.07	−71.79	212.37	−94.69	−19.52	−89.70
B–ClSiF_3_⋯NCNa	−130.90	−83.04	241.07	−108.94	−19.57	−101.38
B–BrSiF_3_⋯NCLi	−104.62	−61.62	186.40	−81.40	−19.93	−81.16
B–BrSiF_3_⋯NCNa	−116.54	−68.45	204.53	−91.58	−20.12	−92.16
B–ISiF_3_⋯NCLi	−95.17	−57.62	176.40	−69.50	−20.62	−66.52
B–ISiF_3_⋯NCNa	−105.16	−61.62	188.03	−76.80	−21.01	−76.55

## Data Availability

Data is available from the authors on request.

## Supplementary Materials

The following supporting information can be downloaded at: https://www.mdpi.com/article/10.3390/molecules28207087/s1, Figure S1: Optimized structure diagram of A–XTF3∙∙∙MCN, marked with the mean of the three angles X–T–F (α, deg), T∙∙∙N distance (R1, Å) and X–T bond length (R2, Å); Figure S2: Linear diagram of interaction energy (Eint) with NOCV orbital energy (E) in the –SiF3; Figure S3: Linear correlation between the interaction energy (Eint) with three attractive terms (Ees, Epol and Edisp) in the –SiF3 complexes.
