# Supplementary material for: The Tetrel Bonds of Hypervalent Halogen Compounds
Source: Molecules. 2023 Oct 14;28(20):7087. doi: 10.3390/molecules28207087 (PMC10609133; doi:10.3390/molecules28207087)
Supplement: Supplementary file 1 [file molecules-28-07087-s001.zip › molecules-2627699-supplementary.pdf]

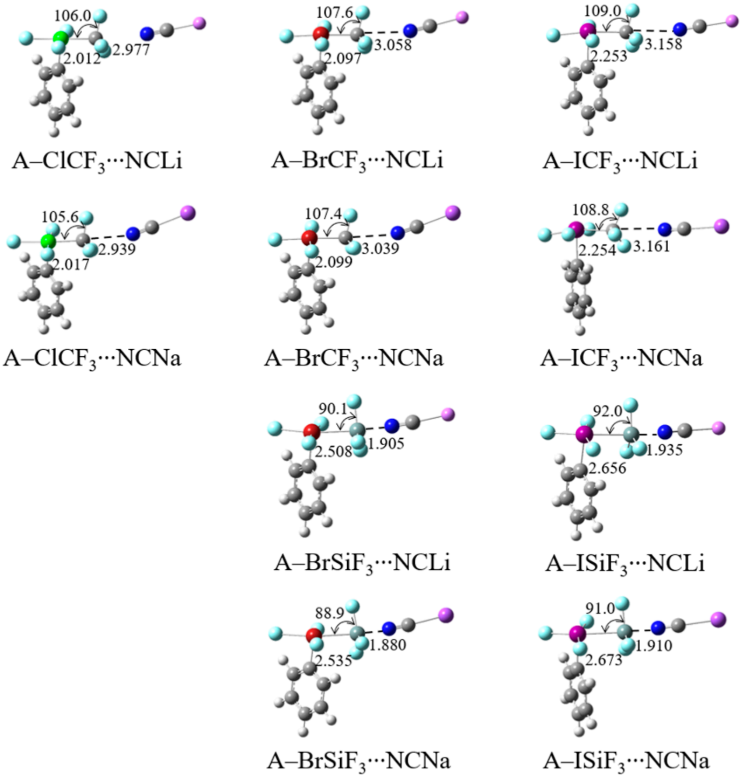

Figure S1. Optimized structure diagram of A–XTF_3···MCN, marked with the mean of the three angles X–T–F (α, deg), T···N distance (R_1, Å) and X–T bond length (R_2, Å).

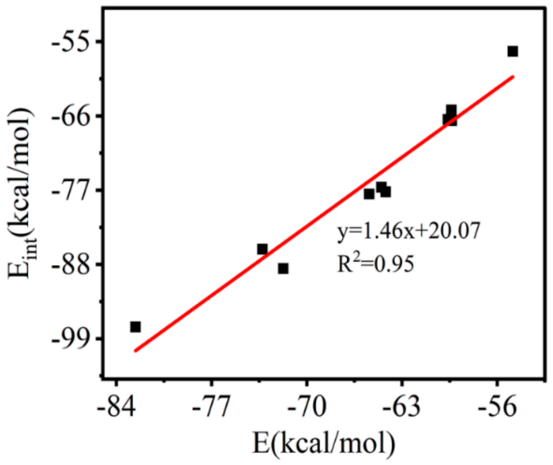

Figure S2. Linear diagram of interaction energy (E_{int}) with NOCV orbital energy (E) in the $–SiF_3$

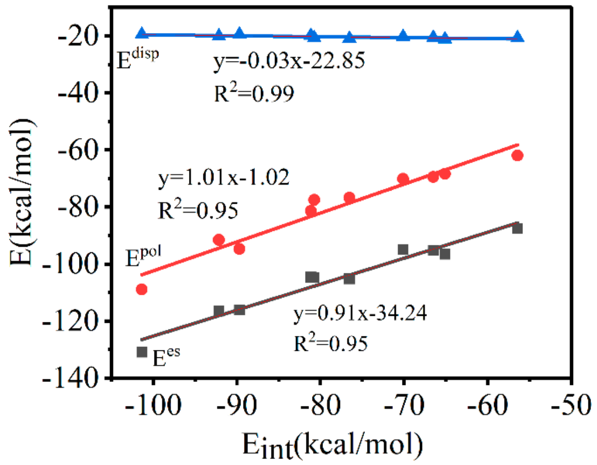

Figure S3. Linear correlation between the interaction energy (E_{int}) with three attractive terms (E^{es}, E^{pol} and E^{disp}) in the –SiF_3 complexes

Cartesian coordinates

A-ClCF3···NCLi

C	2.01988200	2.40637100	0.79087800
C	1.24061300	1.25425500	0.92650100
C	1.54303200	0.22693200	0.06051100
C	2.52782000	0.21055900	-0.90271600
C	3.28457600	1.37770300	-1.00141900
C	3.03116900	2.46647400	-0.16472500
H	1.82768400	3.25149800	1.44889000
H	0.46471400	1.16901700	1.68014200
H	2.70391300	-0.68064900	-1.49637900
H	4.08751200	1.42270300	-1.73482600
H	3.63476600	3.36822300	-0.25415300
C	-1.28696900	-0.38246700	0.02635900
F	-1.63922600	0.02617900	1.21134500
F	-1.16066700	0.59818300	-0.81950100
F	-2.04411100	-1.34840700	-0.41592700
F	0.44626600	-1.14148200	1.91258900
F	0.37922000	-1.33603100	-1.57889000
F	2.28477100	-2.26490900	0.09852300
Cl	0.49301800	-1.30854500	0.16908300
C	-5.11776900	1.14526700	-0.29499900
N	-4.00608300	0.80351800	-0.22503500
Li	-6.96924100	1.68582900	-0.41947700

A-BrCF3···NCLi

C	1.87792400	2.62283900	0.89813200
C	1.19130800	1.41048300	0.99929800
C	1.49068300	0.45726800	0.04579600
C	2.40320200	0.59764300	-0.98225300
C	3.07038900	1.81975500	-1.05135400
C	2.80858600	2.82481000	-0.11882900
H	1.67813100	3.40545000	1.62732200
H	0.47411700	1.22319700	1.79241600
H	2.59323700	-0.21852600	-1.67189100
H	3.80564400	1.97718000	-1.83814300
H	3.33895200	3.77334600	-0.18421600
C	-1.34067600	-0.28260000	0.03693300
F	-1.68837100	0.12617300	1.23696300
F	-1.27069600	0.72241400	-0.80547400
F	-2.15638200	-1.22390000	-0.40000300
Br	0.51841300	-1.24942800	0.11472700
F	0.48322400	-1.07461700	1.95546600
F	0.37130100	-1.18609700	-1.71297400
F	2.35763200	-2.02500800	-0.08110900
C	-5.26093400	1.21833800	-0.29132700
N	-4.13895500	0.93559800	-0.15278900
Li	-7.12503600	1.65274600	-0.54186900

A-ICF$_3$···NCLi

C	1.56030700	2.94749500	0.92285300
C	1.02483900	1.66143600	1.00524400
C	1.43174600	0.74244300	0.04986700
C	2.32454000	1.02169400	-0.97378900
C	2.84293300	2.31474900	-1.03187600
C	2.46296600	3.27041600	-0.08904700
H	1.26515200	3.69459200	1.65763200
H	0.32588400	1.38837000	1.79182200
H	2.61129700	0.25463100	-1.68833500
H	3.55055600	2.57044200	-1.81869600
H	2.87482900	4.27704400	-0.14431200
C	-1.44365800	-0.25846700	-0.02740600
F	-1.84686800	0.14913900	1.17035400
F	-1.43990300	0.75816600	-0.87564200
F	-2.25176500	-1.22198200	-0.47257900
F	0.50231200	-0.91078700	2.06313400
F	0.50827000	-1.11228900	-1.84944300
F	2.56671300	-1.74363600	-0.10052600
I	0.59971700	-1.19767500	0.10703800
C	-5.51781200	1.15111300	-0.25370700

N	-4.38839700	0.86470800	-0.22315400
Li	-7.38597900	1.62198100	-0.31318100

A-ClCF$_3$···NCNa

C	2.33887600	2.46749000	0.83477100
C	1.63977500	1.26355200	0.96074900
C	1.94741200	0.29731300	0.02893700
C	2.86663200	0.38764500	-0.99357900
C	3.54348700	1.60359000	-1.08143400
C	3.28080800	2.63477000	-0.17731400
H	2.14059200	3.26764400	1.54505900
H	0.91897100	1.09683200	1.75434800
H	3.05712500	-0.46285300	-1.63993500
H	4.29280500	1.73234600	-1.86001200
H	3.82244800	3.57567900	-0.25927100
C	-0.84143400	-0.50465200	0.11842400
F	-1.14672200	-0.16237600	1.33453400
F	-0.82065700	0.50737400	-0.69668500
F	-1.54967600	-1.50740500	-0.31873500
F	1.04633500	-1.20972200	1.87973600
F	0.79861400	-1.28266700	-1.60895500
F	2.86648500	-2.13390000	-0.09473400
Cl	1.00886800	-1.30824000	0.12963900
C	-4.76599600	0.61632100	-0.08394200
N	-3.61498600	0.46437000	0.03211200
Na	-6.98950500	0.84381300	-0.36954200

A-BrCF$_3$···NCNa

C	2.05919800	2.72099300	0.91478300
C	1.48372200	1.45179300	1.00944400
C	1.83023900	0.54822700	0.02390900
C	2.68977600	0.78927100	-1.03076600
C	3.24645600	2.06593200	-1.09250200
C	2.93255600	3.02441000	-0.12747500
H	1.81836700	3.46787900	1.66864100
H	0.81336100	1.18658700	1.82111100
H	2.92421800	0.00696800	-1.74553400
H	3.93696700	2.30340200	-1.89951200
H	3.37685400	4.01652200	-0.18747600
C	-0.92856000	-0.43465500	0.10547500
F	-1.25670500	-0.08703900	1.32872800
F	-0.97628700	0.59015200	-0.71257700
F	-1.67283600	-1.43563500	-0.32390900
Br	1.01123200	-1.23642000	0.08614200

F	1.03684900	-1.10625600	1.93137000
F	0.78507100	-1.14716400	-1.73240100
F	2.90530700	-1.84709000	-0.20694800
C	-4.96427200	0.65778300	-0.07041100
N	-3.81097000	0.52802700	0.05111400
Na	-7.18849000	0.81381100	-0.36209900

A-ICF$_3$···NCNa

C	1.44135200	3.07717600	0.97328600
C	1.12489500	1.71936800	1.03615100
C	1.63929100	0.90123200	0.04183400
C	2.43675900	1.34701800	-1.00174400
C	2.73714100	2.70787600	-1.03912200
C	2.24273900	3.56661000	-0.05650600
H	1.05690500	3.74945600	1.73805700
H	0.50894300	1.32001800	1.83792600
H	2.81758100	0.65373600	-1.74671500
H	3.36684000	3.09296700	-1.83932800
H	2.48595100	4.62746100	-0.09380900
C	-1.03011800	-0.55295400	0.03844700
F	-1.44089700	-0.24752000	1.26224100
F	-1.20854200	0.47587100	-0.77484600
F	-1.69816400	-1.61382000	-0.41325200
F	1.07576600	-0.93922100	2.03624600
F	0.95849100	-1.03516800	-1.87957300
F	3.16539900	-1.33013800	-0.22178500
I	1.14278200	-1.15124300	0.06964100
C	-5.24179600	0.43323400	-0.07953400
N	-4.10382300	0.18317200	-0.00929500
Na	-7.44063400	0.84569300	-0.28001900

A-BrSiF$_3$···NCLi

C	2.27587400	2.50328800	0.74515300
C	1.42696700	1.40985200	0.92042200
C	1.62582700	0.33322100	0.07424700
C	2.58561600	0.24499800	-0.91419500
C	3.41404300	1.35931100	-1.06286600
C	3.25991900	2.47782600	-0.24368500
H	2.16751800	3.37193300	1.39264100
H	0.66750500	1.38677300	1.69809100
H	2.68092700	-0.69065400	-1.46732500
H	4.19787900	1.33703200	-1.81847700
H	3.92087300	3.33473200	-0.36856900
F	-1.76645000	0.49351800	1.58740900

F	-1.07426900	1.12409900	-0.97322200
F	-2.36996300	-1.36830200	-0.36649800
F	0.46617600	-0.91723700	2.11027900
F	0.11103900	-1.16545500	-1.58680000
F	2.22701600	-2.36862600	-0.51839600
Si	-1.74075600	0.06369100	0.03620700
Br	0.42106000	-1.18969500	0.24611400
Li	-6.28014700	2.00185900	-1.01722500
N	-3.44572000	0.85894500	-0.26542800
C	-4.48535300	1.30611400	-0.50616400

A-ISiF$_3$···NCLi

C	2.29001600	2.62953600	0.99890400
C	1.58659300	1.43194900	1.14443800
C	1.63119300	0.53357700	0.08842400
C	2.31313400	0.76129700	-1.09807800
C	2.99863100	1.97038700	-1.21836700
C	2.98993800	2.89834400	-0.17617100
H	2.28848100	3.35011000	1.81552200
H	1.03873400	1.19626300	2.05294000
H	2.31714100	0.00093800	-1.87552400
H	3.55190800	2.17928200	-2.13279400
H	3.53603700	3.83528600	-0.27916400
F	-1.83156200	0.56792200	1.58493300
F	-1.14057400	1.19738800	-0.99679300
F	-2.53379800	-1.25878600	-0.39310600
F	0.44771600	-0.88356200	2.20753800
F	-0.01226300	-1.15312500	-1.74174200
F	2.19372200	-1.99822600	-0.89606200
I	0.47975600	-1.24031200	0.19683900
Si	-1.79186500	0.12634600	0.02777800
C	-4.49903500	1.54647800	-0.43204400
N	-3.49071500	1.01235200	-0.24157800
Li	-6.20342700	2.48180900	-0.81228500

A-BrSiF$_3$···NCNa

C	2.29521000	2.70137900	0.76879500
C	1.59567100	1.50435600	0.92066000
C	1.91021600	0.48706500	0.03598700
C	2.85620800	0.54976500	-0.96758200
C	3.53354900	1.76550700	-1.09260300
C	3.25506500	2.83088300	-0.23620200
H	2.08964900	3.52939900	1.44521900
H	0.86159400	1.36140600	1.70943600

H	3.06109100	-0.35648900	-1.54335000
H	4.29917600	1.86721800	-1.86005600
H	3.79822500	3.76902600	-0.34417300
F	-1.38780400	0.14280900	1.72347600
F	-0.94568300	0.94208200	-0.84462800
F	-1.85081400	-1.72102700	-0.26789800
F	1.03717900	-0.98515800	2.06209400
F	0.51136500	-1.12479800	-1.61993200
F	2.83857100	-2.10418000	-0.73788500
Si	-1.43089500	-0.21984600	0.15581800
Br	0.91555200	-1.17786800	0.19163400
Na	-6.55754000	1.14085600	-0.60077900
N	-3.21901500	0.33534100	-0.01213800
C	-4.32605400	0.64243300	-0.16892700

A-ISiF$_3$···NCNa

C	2.21977400	2.87790400	1.05892400
C	1.72130000	1.57808200	1.17453400
C	1.82843100	0.75298800	0.06511500
C	2.37818100	1.14688100	-1.14646500
C	2.85801300	2.45329900	-1.23566700
C	2.78317300	3.31370100	-0.13915600
H	2.16764700	3.54646600	1.91704700
H	1.28407100	1.21091200	2.09936500
H	2.44321900	0.43732100	-1.96762300
H	3.30348900	2.79379400	-2.16942900
H	3.17080600	4.32862000	-0.21895100
F	-1.53434100	0.05905600	1.72876700
F	-1.08492300	0.96959700	-0.82909700
F	-1.98076100	-1.73642700	-0.34302200
F	1.01755900	-0.93939200	2.16202500
F	0.39254400	-1.09952400	-1.77657700
F	2.75199300	-1.59264200	-1.08809400
I	0.99287400	-1.19150600	0.13259400
Si	-1.51036200	-0.25542400	0.14098400
C	-4.42816800	0.65543300	-0.13029200
N	-3.32809900	0.31059800	-0.00982400
Na	-6.61569700	1.32897400	-0.46916900

B-ClCF$_3$···NCLi

C	-2.68495900	1.97919800	-1.20481100
C	-2.06508600	0.73317700	-1.09679200
C	-1.30325600	0.55416300	0.04352200
C	-1.09506600	1.44475200	1.08118100

C	-1.73527200	2.68034100	0.93016300
C	-2.53107800	2.94828100	-0.19707800
H	-3.31784100	2.17789300	-2.07234300
H	-2.19749400	-0.05656500	-1.83743500
H	-0.50773800	1.20404300	1.95613100
H	-1.61798800	3.42384900	1.71897200
H	-3.02465800	3.90462400	-0.30031900
Cl	-2.83615900	-2.17237000	-0.06834800
C	1.51420900	-0.33926400	0.16275700
F	1.49030800	0.63460000	-0.68961600
F	1.77173500	0.02657200	1.36754600
F	2.16615300	-1.37968000	-0.23120500
F	-0.18782400	-1.11952800	-1.55775400
F	-0.46345700	-0.99869900	1.92876600
Cl	-0.42883600	-1.09742900	0.17647200
C	5.32278800	0.67653800	-0.43757200
N	4.20575100	0.52406400	-0.04605200
Li	7.14482300	0.90059800	-1.28286900

B-BrCF_3···NCLi

C	-2.64294100	-1.64369200	0.92312200
C	-1.36201000	-1.09088400	1.01821700
C	-1.04263500	-0.15457300	0.05671300
C	-1.85558100	0.28680900	-0.96807000
C	-3.12427200	-0.28778900	-1.02592300
C	-3.51324600	-1.24624800	-0.08896500
H	-2.95170700	-2.38486000	1.65793900
H	-0.67556400	-1.36971100	1.81098900
H	-1.52694800	1.05195300	-1.66514400
H	-3.81043900	0.02959600	-1.80869900
H	-4.50889400	-1.68276900	-0.14669300
Cl	-0.37237500	2.88852400	0.29371400
C	1.64541800	-1.25358900	-0.24059600
F	1.71270100	-1.95641800	0.87258800
F	0.94800400	-1.90439300	-1.14873700
F	2.85919800	-0.98853900	-0.69044800
Br	0.77388300	0.64477700	0.11618400
F	0.83908900	0.26391700	1.91421900
F	0.76207800	0.69915300	-1.72039500
C	3.26977310	-5.25934784	-1.06576672
N	2.79816804	-4.17905079	-0.85462803
Li	4.03952237	-7.02259835	-1.41038528

B-ICF_3···NCLi

C	-2.91510100	-1.48592800	0.99068400
C	-1.60928000	-0.99580600	1.07146800

C	-1.19986200	-0.14996900	0.05408400
C	-1.98316500	0.24087200	-1.02061900
C	-3.28039600	-0.26596300	-1.06995700
C	-3.74213800	-1.12354700	-0.07044500
H	-3.27990800	-2.15110900	1.77115500
H	-0.95582900	-1.25382400	1.89986600
H	-1.60414700	0.92007100	-1.78004900
H	-3.93038300	0.01974400	-1.89481800
H	-4.75897700	-1.50978100	-0.11850800
Cl	-0.31730800	2.91960800	0.21984900
C	1.47318100	-1.52964800	-0.24937100
F	1.33634500	-2.27427000	0.84385500
F	0.78953300	-2.08687400	-1.24181000
F	2.76651600	-1.48402600	-0.58186200
F	0.79282300	0.13253700	2.02121200
F	0.72756700	0.62714000	-1.85946800
I	0.78771700	0.60322500	0.10401000
C	2.59014352	-5.71513831	-1.21391460
N	2.31598807	-4.60794551	-0.97006318
Li	3.04329275	-7.54520796	-1.61697457

B-ClCF$_3$···NCNa

C	-2.98890400	-0.33268200	0.98307800
C	-1.75772400	0.31971700	0.91780300
C	-0.90659000	-0.14435500	-0.05914400
C	-1.12917800	-1.15420800	-0.96707300
C	-2.37485200	-1.78209600	-0.85879700
C	-3.29258800	-1.37529300	0.10603900
H	-3.71446800	-0.00400300	1.72447300
H	-1.49283500	1.15815200	1.55421500
H	-0.40900700	-1.43090300	-1.72954200
H	-2.61566700	-2.58800600	-1.54917600
H	-4.26003000	-1.87004300	0.17184800
Cl	-0.45422900	2.90829600	-0.36217500
C	1.83834400	-0.98769100	0.22553500
F	1.21105200	-1.70236500	1.12081400
F	1.99314000	-1.65347100	-0.88920900
F	2.97820700	-0.52834700	0.67347600
F	0.79638900	0.81248700	1.61815100
F	0.87297400	0.40302500	-1.84376100
Cl	0.77747000	0.70891000	-0.13027800
C	4.03491519	-4.78622633	0.78049449
N	3.43035221	-3.80357595	0.59899708
Na	5.19533896	-6.67236704	1.12886831

B-BrCF$_3$···NCNa

C	-2.64294100	-1.64369200	0.92312200
C	-1.36201000	-1.09088400	1.01821700
C	-1.04263500	-0.15457300	0.05671300
C	-1.85558100	0.28680900	-0.96807000
C	-3.12427200	-0.28778900	-1.02592300
C	-3.51324600	-1.24624800	-0.08896500
H	-2.95170700	-2.38486000	1.65793900
H	-0.67556400	-1.36971100	1.81098900
H	-1.52694800	1.05195300	-1.66514400
H	-3.81043900	0.02959600	-1.80869900
H	-4.50889400	-1.68276900	-0.14669300
Cl	-0.37237500	2.88852400	0.29371400
C	1.64541800	-1.25358900	-0.24059600
F	1.71270100	-1.95641800	0.87258800
F	0.94800400	-1.90439300	-1.14873700
F	2.85919800	-0.98853900	-0.69044800
Br	0.77388300	0.64477700	0.11618400
F	0.83908900	0.26391700	1.91421900
F	0.76207800	0.69915300	-1.72039500
C	3.33576434	-5.30225765	-1.04823905
N	2.88172137	-4.20778335	-0.86499280
Na	4.20407257	-7.39532178	-1.39867776

B-ICF$_3$···NCNa

C	-2.91510100	-1.48592800	0.99068400
C	-1.60928000	-0.99580600	1.07146800
C	-1.19986200	-0.14996900	0.05408400
C	-1.98316500	0.24087200	-1.02061900
C	-3.28039600	-0.26596300	-1.06995700
C	-3.74213800	-1.12354700	-0.07044500
H	-3.27990800	-2.15110900	1.77115500
H	-0.95582900	-1.25382400	1.89986600
H	-1.60414700	0.92007100	-1.78004900
H	-3.93038300	0.01974400	-1.89481800
H	-4.75897700	-1.50978100	-0.11850800
Cl	-0.31730800	2.91960800	0.21984900
C	1.47318100	-1.52964800	-0.24937100
F	1.33634500	-2.27427000	0.84385500
F	0.78953300	-2.08687400	-1.24181000
F	2.76651600	-1.48402600	-0.58186200
F	0.79282300	0.13253700	2.02121200
F	0.72756700	0.62714000	-1.85946800

I	0.78771700	0.60322500	0.10401000
C	2.56754347	-5.70223574	-0.96147656
N	2.31340776	-4.57586193	-0.78615814
Na	3.05534230	-7.86424530	-1.29799016

B-ClSiF_3···NCLi

C	1.72083800	2.97637700	0.75192500
C	1.05369700	1.76767000	0.95724400
C	1.36122500	0.74097400	0.08320900
C	2.26250500	0.80191400	-0.96020200
C	2.91049700	2.02687800	-1.13369900
C	2.64185400	3.10308400	-0.28795600
H	1.52176100	3.81349200	1.41879000
H	0.35345400	1.62750200	1.77589200
H	2.48002400	-0.08384400	-1.55566700
H	3.64602700	2.12405100	-1.93007400
H	3.16263800	4.04839300	-0.43410900
Cl	3.04503100	-2.20886800	-0.31422600
F	-2.03991500	0.34802600	1.54272300
F	-1.42599900	0.98446300	-1.03929100
F	-2.29473500	-1.63103500	-0.36512500
F	0.41227900	-0.63382700	2.15978800
F	0.21722900	-1.07348200	-1.51741500
Si	-1.97943700	-0.09859300	0.00295300
C	-4.85303800	0.68861600	-0.56500400
N	-3.75945400	0.39814800	-0.32176200
Li	-6.73129300	1.18630800	-1.05867200
Cl	0.51848729	-0.78880152	0.30041860

B-BrSiF_3···NCLi

C	-2.18636800	-2.27909400	0.91657000
C	-1.14742500	-1.35489900	1.06778900
C	-1.09557200	-0.36252800	0.11026100
C	-1.94378700	-0.19057500	-0.96325900
C	-2.96393800	-1.13542900	-1.07443200
C	-3.08264500	-2.16917800	-0.14375400
H	-2.28726400	-3.07959600	1.64663800
H	-0.45140000	-1.40364100	1.90024400
H	-1.83198100	0.64464200	-1.64915900
H	-3.67299000	-1.04753100	-1.89517100
H	-3.88692500	-2.89543700	-0.24593800
Cl	-1.44155400	2.81720400	0.04958700
F	2.22997700	-1.59725300	1.10600300
F	1.32172800	-1.80373800	-1.32621200

F	3.24167100	-0.07459800	-0.77035300
Br	0.39530600	0.95802200	0.23976400
F	0.49610100	0.61340700	2.05135200
F	0.53778000	0.91500100	-1.62140600
Si	1.91959800	-0.79763100	-0.24463100
C	4.62416079	-3.86159840	-1.27146590
N	3.88031419	-3.00068565	-1.01451824
Li	5.85365858	-5.28459383	-1.69617253

B-$ISiF_3$···NCLi

C	-2.22066300	-2.43571300	0.99896700
C	-1.24151900	-1.44620300	1.12656900
C	-1.17423500	-0.51250600	0.10550600
C	-1.98488800	-0.48918000	-1.01732700
C	-2.94661000	-1.49372500	-1.11225200
C	-3.06329900	-2.45869700	-0.11037700
H	-2.31897900	-3.18555400	1.78173100
H	-0.58475600	-1.40344400	1.99145100
H	-1.87986800	0.28338200	-1.77489500
H	-3.60895100	-1.51296300	-1.97555700
H	-3.82099600	-3.23575200	-0.19619000
Cl	-1.66888800	2.68391800	-0.02593100
F	2.20880100	-1.81683700	1.08521000
F	1.40284200	-1.91456100	-1.38941900
F	3.38941300	-0.34175800	-0.72166700
F	0.49425200	0.53153600	2.12154700
F	0.37945100	0.96525900	-1.78101700
I	0.33545400	0.99850700	0.19418100
Si	1.98000400	-0.94926600	-0.24683700
C	4.64024806	-4.09537358	-0.92617541
N	3.90624477	-3.21155548	-0.72466775
Li	5.85347593	-5.55622908	-1.25924574

B-$ClSiF_3$···NCNa

C	1.90861000	3.04815500	0.74621200
C	1.32729300	1.79816700	0.96337400
C	1.65372700	0.80187300	0.06072000
C	2.50247000	0.93136300	-1.02026300
C	3.06599100	2.19629300	-1.20466800
C	2.77087500	3.24402900	-0.33279600
H	1.69147700	3.86346000	1.43418800
H	0.67910700	1.60332500	1.81352100
H	2.75003600	0.06617600	-1.63496800
H	3.75910000	2.34665400	-2.03037100

H	3.22683100	4.22107400	-0.48748500
Cl	3.50804200	-2.05115600	-0.53419900
F	-1.64492800	0.15043200	1.70411200
F	-1.21340100	0.88874900	-0.88248100
F	-1.86558500	-1.79409000	-0.23378800
F	0.92341100	-0.66518700	2.16368900
F	0.52574100	-1.04993600	-1.50667600
Si	-1.67127400	-0.24608400	0.15050100
C	-4.60372500	0.31688400	-0.25147300
N	-3.47466100	0.12245600	-0.07002800
Na	-6.87753900	0.62419000	-0.71784600
Cl	0.91847857	-0.77963034	0.29706071

B-BrSiF$_3$···NCNa

C	-2.18636800	-2.27909400	0.91657000
C	-1.14742500	-1.35489900	1.06778900
C	-1.09557200	-0.36252800	0.11026100
C	-1.94378700	-0.19057500	-0.96325900
C	-2.96393800	-1.13542900	-1.07443200
C	-3.08264500	-2.16917800	-0.14375400
H	-2.28726400	-3.07959600	1.64663800
H	-0.45140000	-1.40364100	1.90024400
H	-1.83198100	0.64464200	-1.64915900
H	-3.67299000	-1.04753100	-1.89517100
H	-3.88692500	-2.89543700	-0.24593800
Cl	-1.44155400	2.81720400	0.04958700
F	2.22997700	-1.59725300	1.10600300
F	1.32172800	-1.80373800	-1.32621200
F	3.24167100	-0.07459800	-0.77035300
Br	0.39530600	0.95802200	0.23976400
F	0.49610100	0.61340700	2.05135200
F	0.53778000	0.91500100	-1.62140600
Si	1.91959800	-0.79763100	-0.24463100
C	4.62212063	-3.94523344	-1.19524257
N	3.87400669	-3.08679729	-0.93552662
Na	6.05808218	-5.59295212	-1.69375235

B-ISiF$_3$···NCNa

C	1.78312200	3.20395000	0.90277400
C	1.31769600	1.90022400	1.08691700
C	1.57708100	0.98984300	0.07462000
C	2.24970800	1.28432200	-1.09961400
C	2.69813000	2.59655800	-1.25526000
C	2.46926800	3.54841800	-0.26104000

H	1.61370600	3.94571600	1.68167500
H	0.79747800	1.60008000	1.99285200
H	2.44614400	0.50790800	-1.83608200
H	3.24717400	2.86509800	-2.15614000
H	2.83715200	4.56537400	-0.39088600
Cl	3.48707100	-1.70118100	-0.74563700
F	-1.79442200	0.13697800	1.67991500
F	-1.33112900	0.92800300	-0.92749800
F	-2.02704500	-1.76534900	-0.34598300
F	0.84727200	-0.60855400	2.27217600
F	0.52581600	-1.05096300	-1.65933100
I	0.85758400	-0.99354000	0.29729500
Si	-1.74497900	-0.23526300	0.10972700
C	-4.69878900	0.38383600	-0.26533600
N	-3.57073100	0.16926200	-0.10432700
Na	-6.96461100	0.72712800	-0.67727200
